# The demanding grey zone: Sport indices by cardiac magnetic resonance imaging differentiate hypertrophic cardiomyopathy from athlete’s heart

**DOI:** 10.1371/journal.pone.0211624

**Published:** 2019-02-14

**Authors:** Csilla Czimbalmos, Ibolya Csecs, Attila Toth, Orsolya Kiss, Ferenc Imre Suhai, Nora Sydo, Zsofia Dohy, Astrid Apor, Bela Merkely, Hajnalka Vago

**Affiliations:** Heart and Vascular Center, Semmelweis University, Budapest, Hungary; University of California, Davis, UNITED STATES

## Abstract

**Background:**

We aimed to characterize gender specific left ventricular hypertrophy using a novel, accurate and less time demanding cardiac magnetic resonance (CMR) quantification method to differentiate physiological hypertrophy and hypertrophic cardiomyopathy based on a large population of highly trained athletes and hypertrophic cardiomyopathy patients.

**Methods:**

Elite athletes (n = 150,>18 training hours/week), HCM patients (n = 194) and athletes with hypertrophic cardiomyopathy (n = 10) were examined by CMR. CMR based sport indices such as maximal end-diastolic wall thickness to left ventricular end-diastolic volume index ratio (EDWT/LVEDVi) and left ventricular mass to left ventricular end-diastolic volume ratio (LVM/LVEDV) were calculated, established using both conventional and threshold-based quantification method.

**Results:**

Whereas 47.5% of male athletes, only 4.1% of female athletes were in the grey zone of hypertrophy (EDWT 13-16mm). EDWT/LVEDVi discriminated between physiological and pathological left ventricular hypertrophy with excellent diagnostic accuracy (AUC_CQ_:0.998, AUC_TQ_:0.999). Cut-off value for LVM/LVEDV_CQ_<0.82 mm×m^2^/ml and for EDWT/LVEDVi_TQ_<1.27 discriminated between physiological and pathological left ventricular hypertrophy with a sensitivity of 77.8% and 89.2%, a specificity of 86.7% and 91.3%, respectively. LVM/LVEDV evaluated using threshold-based quantification performed significantly better than conventional quantification even in the male subgroup with EDWT between 13-16mm (p<0.001).

**Conclusions:**

Almost 50% of male highly trained athletes can reach EDWT of 13 mm. CMR based sport indices provide an important tool to distinguish hypertrophic cardiomyopathy from athlete’s heart, especially in highly trained athletes in the grey zone of hypertrophy.

## Introduction

Regular and intensive physical training triggers structural and functional changes of the heart, including increased left ventricular wall thickness and cardiac mass, contributing to a clinical challenge of differentiating between athlete’s heart and hypertrophic cardiomyopathy (HCM) [1–3]. Relevance of this question arises from the fact that HCM is one of the most common causes of sudden unexpected cardiac death in young competitive athletes [4, 5]. Furthermore, false positive diagnosis of HCM may lead to unnecessary interruption of a professional sporting career.

Based on large cohort studies, the physiological upper limit for maximal end-diastolic wall thickness (EDWT) is 16 mm, athletes with a maximal EDWT >16 mm may be considered to have pathological left ventricular hypertrophy [3, 6]. According to echocardiographic literature, about 2% of highly trained athletes fall into the grey zone by having interventricular septal wall thickness between 12 and 15 mm [3]. According to recent cardiac magnetic resonance (CMR) data phenotypic crossover between extreme forms of the athlete’s heart and mild forms of pathological hypertrophy is much more common than former literature suggested, the number can reach 23% among healthy young army recruits [7]. Although ventricular septal to free wall thickness ratio in athletes is usually normal (<1.3), occasionally athletes may show mild asymmetric thickening of the anterior basal septum [8], recent scientific data suggest that intensive physical training may also increase the degree of left ventricular asymmetry escalating difficulties in differentiation between HCM and athlete’s heart [4]. Additional echocardiographic data i.a. diastolic dysfunction or strain parameters may also add important information to the diagnostic work-up [9–11]. Structural and functional cardiac parameters and certain geometric indices determined by CMR can improve the sensitivity and specificity for differentiating physiological left ventricular hypertrophy from HCM [12]. Although it is well known that left ventricular hypertrophy is less pronounced in female elite athletes, data available about gender-specific normal left ventricular values for elite professional athletes determined by CMR are very limited [13, 14]. Additionally, the image analysis protocol may have a high impact on ventricular mass and volumetric data [15]. Evaluation of trabeculae and papillary muscles can fundamentally change left ventricular parameters due to the large papillary muscles and pronounced trabeculation, especially in HCM patients [16, 17]. Recently, a semi-automatic segmentation algorithm for CMR-derived mass, volume and function measurement became available. Using this algorithm the papillary muscles and trabeculae are excluded from the blood pool. Compared to the conventional quantification, this semi-automatic quantification method for CMR-derived mass, volume and function measurement enables a more accurate and rapid quantification [18, 19].

Our goal was to characterize gender specific left ventricular (LV) hypertrophy including sport indices for highly trained professional athletes and HCM patients with conventional and threshold-based quantification method. We aimed to determine accurate CMR cut-off values based on a large population which can help to distinguish physiological left ventricular hypertrophy from HCM in both gender. We tested whether threshold-based quantification (TQ) method is useful in the diagnostic workup in the differentiation of physiological and pathological left ventricular hypertrophy especially in athletes in the grey zone of hypertrophy.

## Materials and methods

### Study participants

Our prospective observational study was conducted in the Heart and Vascular Center of Semmelweis University between 2011 and 2015. The total study population of nonathlete HCM patients (n = 194, 50.2±13.6y, 108 male, body surface area (BSA) = 2±0.17 m^2^), healthy athletes (n = 150, 24.2±4.8y, 101 male, BSA = 1.92±0.19 m^2^) and athletes with HCM (n = 10, 31±10y; 9 male, BSA = 1.94±0.19 m^2^) were examined by CMR. Ethical approval was obtained from the Central Ethics Committee of Hungary, informed consent was obtained from all individual participants included in the study. HCM patients with ejection fraction>50% were consecutively enrolled. HCM was defined according to the current ESC guideline [20], as a maximal wall thickness ≥15 mm measured by CMR that is not explained solely by loading conditions, in 11% of our HCM patients who had wall thickness between 13–14 mm we evaluated other features including family history, ECG, typical LGE pattern. Elite athletes with a minimum of 18 hours training per week for at least the last 18 months performing highly dynamic and at least moderately static sports were consecutively recruited, male and female athletes with an average of 22.1±5.1 and 21.2±3.5 hours training per week, respectively. Elite athletes, all of them members of the Hungarian National or Olympic Team, free of any cardiovascular diseases with no ECG abnormality suggesting structural heart disease and without systolic or diastolic dysfunction on echocardiography were enrolled. Fifty-one percent of the athletes are Olympic, World Championship and/or European Championship medallist. Male athlete cohort was comprised from canoe and kayak paddlers (n = 46), water-polo players (n = 31) and rowers (n = 24). Sixty-seven percent of female athletes comprising canoe and kayak paddlers, water-polo players and rowers, additional athletes (handball, speed skating, swimming, athletics, tennis, cross country skiing, basketball or cycling) were also examined. Ten additional athletes (14.4±6.5 training hours/week) were examined using CMR during training or competition period because of the suspicion of HCM, where the comprehensive investigation confirmed the diagnosis.

### CMR examination

CMR examinations were conducted on a 1.5 T MR scanner (Achieva, Philips Medical Systems) with a 5-channel cardiac coil. Retrospectively-gated, balanced steady-state free precession (bSSFP) segmented cine images were acquired in 2-chamber, 4-chamber and LV outflow tract views. Additionally, short-axis images with full coverage of the left ventricle were obtained. Slice thickness was 8 mm with no interslice gap, field of view 350 mm on average adapted to body size. Late gadolinium enhancement (LGE) imaging was performed in 253 cases after the patients gave their informed consent (71% of athletes and 98% of HCM patients). During an inspiratory breath-hold, a bolus of gadobutrol (0.15 mmol/kg) was injected at a rate of 2–3 ml/s through antecubital intravenous line. Peripheral bolus injection was performed with a MR-conditional power injector followed by saline flush. Contrast-enhanced images were acquired using a segmented inversion recovery sequence with additional phase sensitive reconstructions in the same views used for cine images 10–20 min after contrast administration.

### Image analysis

**Fig 1:**
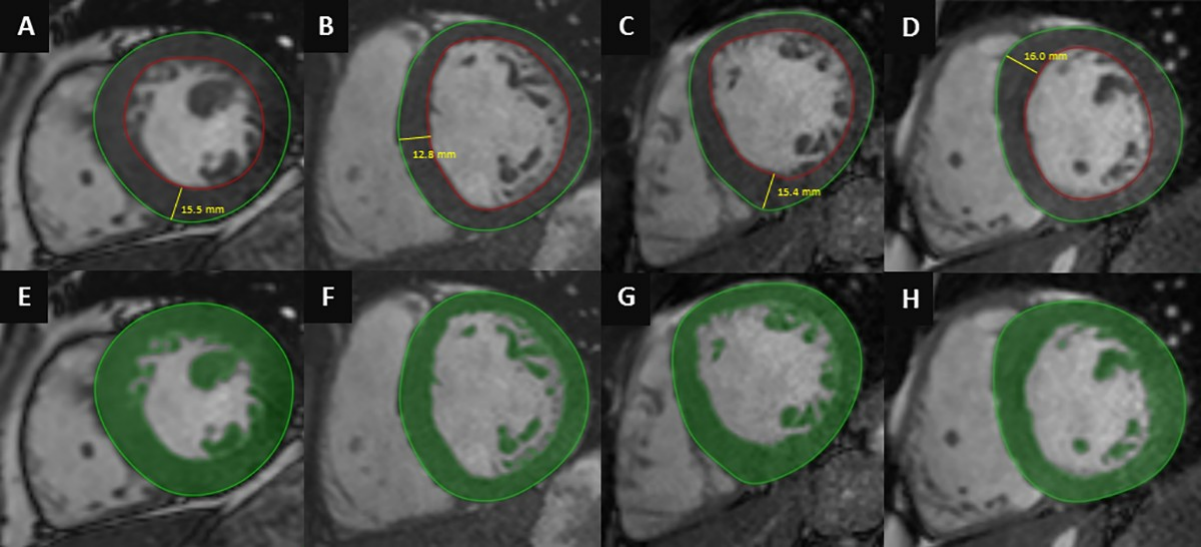
Conventional quantification (A,B,C,D) and threshold-based quantification method (E,F,G,H) in HCM (A,E), healthy athlete (B,F), healthy athlete with EDWT in the grey zone of hypertrophy (C,G) and athlete with HCM (D,H).

All images were evaluated with Medis QMass 7.6 quantification software (Medis Medical Imaging Software, Leiden, The Netherlands). Endocardial and epicardial contour detection was performed by a blinded expert observer (>5000 original CMR cases) manually on short axis cine images. Quantification of the left ventricular ejection fraction (LVEF), end-systolic volume (LVESV), end-diastolic volume (LVEDV), stroke volume (LVSV) and myocardial mass (LVM) were performed using two different quantification methods: conventional quantification method (CQ) (Medis QMass 7.6) and threshold-based quantification method (TQ) (Medis QMass 7.6 MassK algorithm). **(Fig 1)** Using TQ the trabeculae and papillary muscles (TPM) were also quantified, the semi-automatic threshold-based algorithm allows to estimate the spatially varying signal intensities of blood and muscle within an observer-provided epicardial contour. Voxels with signal intensities above the specified threshold are considered to be blood, voxels with signal intensities below the threshold are considered to be muscle. For the analysis, the SI threshold was set to 50% invariably. Left ventricular volumes and masses were standardized to body surface area (BSA)–LVESVi, LVEDVi, LVSVi, LVMi, TPMi. Maximal end-diastolic wall thickness (EDWT) measurements were taken in a short axis slice perpendicularly to the myocardial center line excluding right ventricular trabeculation. Minimal EDWT was measured in the same slice as the maximal EDWT. To characterize the left ventricular hypertrophy, sport indices were derived using both conventional and threshold-based quantification method, such as left ventricular maximal diastolic wall thickness to end-diastolic volume index ratio (EDWT(mm)/LVEDVi(ml/m^2^)) and left ventricular mass to end-diastolic volume ratio (LVM(g)/LVEDV(ml)). Using threshold-based quantification TPM% (TPM% (TPM(g)/LVM(g)*100) was also established.

### Statistical analysis

All continuous variables are expressed as mean ± standard deviation, categorical variables are expressed as percentages. Between-groups comparisons of CMR parameters and sport indices were based on least-squares linear regression if normality assumptions were satisfied, and median regression otherwise. Models were stratified by HCM when comparing males versus females, and stratified for sex when comparing HCM patients versus athletes (i.e., practically, pairwise comparisons were made). Adjustment for age and heart frequency was applied by including these terms in the models as explanatory variables. Diagnostic accuracy of CMR parameters was evaluated using receiver operating characteristic (ROC) curve analysis. Cut-off values of sport indices for HCM patient versus athlete classification were set to maximize the proportion of subjects correctly classified. P-values <0.05 were considered to indicate statistical significance.

## Results

### Baseline characteristics

#### Healthy athletes

None of our healthy athletes had positive family history of sudden cardiac death or HCM, all of them were asymptomatic. Seventy percent of athletes showed J-point elevation, 27% Sokolow or Cornell index positivity. Three athletes had T-wave inversion in more than one contiguous leads, all of them in V1-V3 leads without signs of left ventricular hypertrophy. None of the athletes showed pathological Q wave or ST-depression, and none of them showed any LGE (n = 108).

#### HCM patients

Twenty-eight percent and 30% of our HCM patients had positive family history for sudden cardiac death or HCM, respectively. Twenty-seven percent of HCM patients reported syncope and 32% palpitation. Only 55% of HCM patients showed positive Sokolow or Cornell index, 82% showed T-wave inversion. Seventy-five percent (n = 143) of HCM patients demonstrated LGE, mainly in the hypertrophic segments (80%) localized to the septum and anterior wall, in the right ventricular insertion points (55%), or only mild enhancement in the hypertrophic segments (19%). In our study population LGE showed excellent positive predictive value ($PPV=\frac{143}{143+0}*100=100\%$) but low predictive value ($NPV=\frac{108}{108+47}*100=70\%$).

#### Athletes with HCM

**Fig 2:**
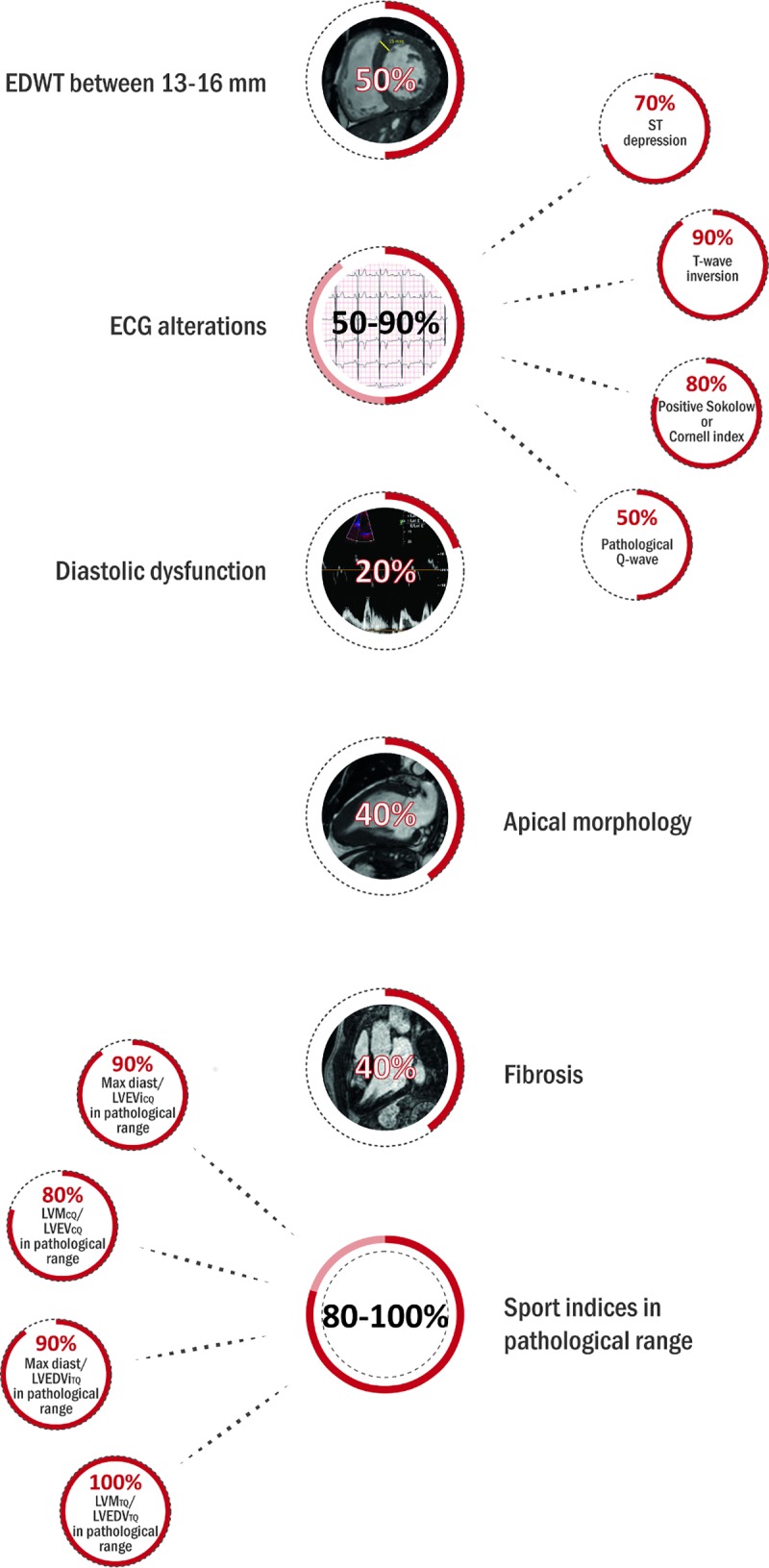
The clinical characteristics of athletes with HCM including pathological ECG findings, diastolic dysfunction evaluated using echocardiography and EDWT, morphology, fibrosis and sport indices evaluated using CMR.

**Fig 3:**
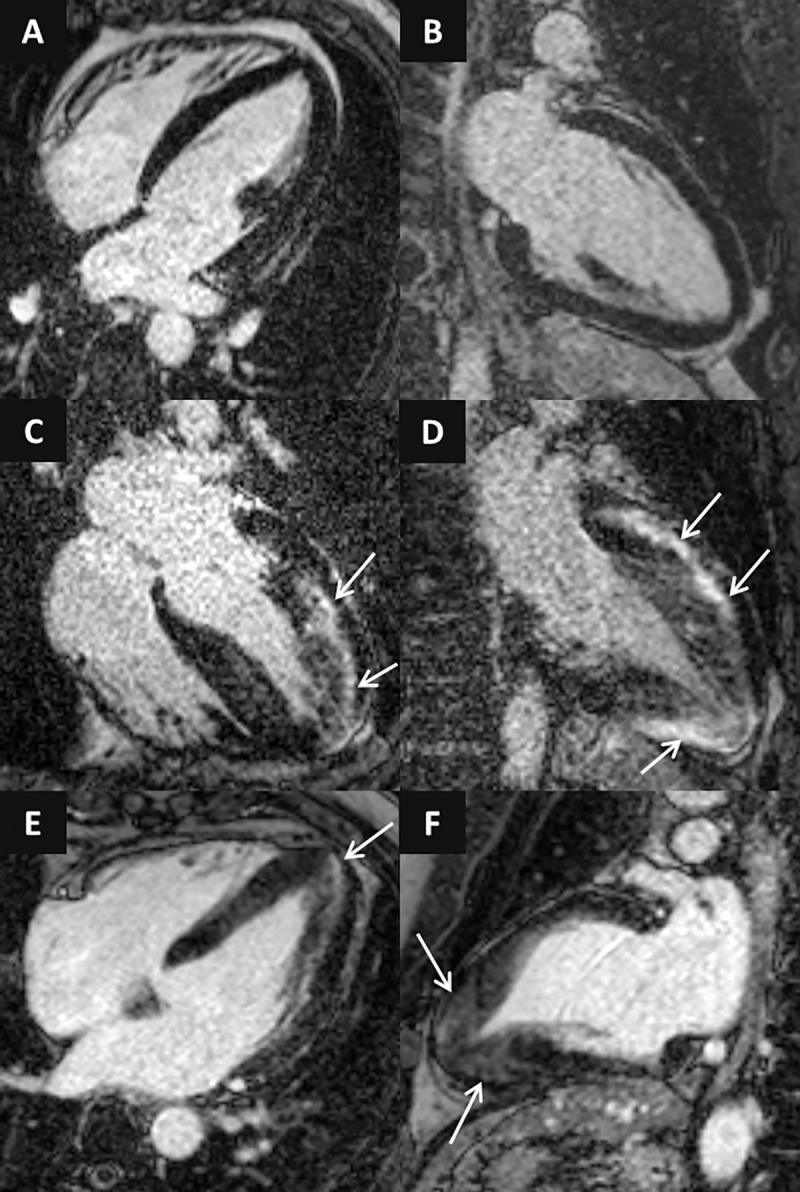
LGE images in 4-chamber (A,C,E) and 2-chamber views (B,D,F) of athletes with HCM. Concentric HCM with no LGE (A,B), asymmetric form with pronounced midmyocardial patchy LGE (C,D) and apical HCM with mild LGE in the hypertrophic segments (D-F).

Ten additional athletes (31±10y; 9 male, 14.4±6.5 training hours/week, football (n = 3), basketball (n = 2), snowboard (n = 1), pentathlon (n = 1), kayak (n = 1), water-polo (n = 1), wrestling (n = 1)) were examined with CMR during training or competition period because of the suspicion of HCM, where the comprehensive investigation including resting ECG, ambulatory ECG monitoring, echocardiography, CMR, patient and family history confirmed the diagnosis. No secondary cause for left ventricular hypertrophy was observed, and after training cessation for 3 months only blunted, or no deconditioning effect was observed, left ventricular mass and wall-thickness remained in the pathological range. EDWT measured by CMR was 17.7 ± 2.7 mm, five of them showed EDWT between 13–16 mm. Left ventricular CMR parameters of athletes with HCM are present in **Table 1**. All of them showed at least one pathological ECG finding according to the 2014 ESC Guidelines on diagnosis and management of hypertrophic cardiomyopathy (15), 8 patients had positive Sokolow or Cornell index and 9 patients showed T wave inversion on 12-lead ECG. Only two patients showed diastolic dysfunction on echocardiography. **(Fig 2)** Apical HCM morphology was confirmed in four patients. Only four patients showed LGE in the hypertrophic regions and/or in the right ventricular insertion points. **(Fig 3)**

**Table 1:** Left ventricular CMR parameters of athletes with HCM using conventional (CQ) and threshold-based (TQ) quantification.

	Athletes with HCM (n = 10)
	mean ± SD
EDWT (mm)	17.7 ± 2.7
Max/min EDWT	2.28 ± 0.52
Conventional quantification	
LVEF_CQ_ (%)	63.3 ± 4.2
LVESVi_CQ_ (ml/m^2^)	37.3 ±5.1
LVEDVi_CQ_ (ml/m^2^)	101.3 ±7.8
LVSVi_CQ_ (ml/m^2^)	64.1 ±6.5
LVMi_CQ_ (g/m^2^)	94.8 ±15.0
max diast/LVEDVi_CQ_ (mm×m^2^×ml)	0.18 ±0.03
LVM_TQ_/LVEDV_CQ_ (g/ml)	0.94 ±0. 13
Threshold-based quantification	
EF_TQ_ (%)	72.3 ± 4.6
LVESVi_TQ_ (ml/m^2^)	21.1 ± 4.3
LVEDVi_TQ_ (ml/m^2^)	75.8 ± 7.7
LVSVi_TQ_ (ml/m^2^)	54.7 ± 6.1
LVMi_TQ_ (g/m^2^)	121.3 ± 16.9
max diast/LVEDVi_TQ_ (mm×m^2^×ml)	0.24 ±0.05
LVM_TQ_/LVEDV_TQ_ (g/ml)	1.62 ±0.28
Trab M (g)	41.1 ±12.6
Trab Mi (g/m^2^)	21.4 ±6.3
Trab % (%)	17.4 ± 4.4

Abbreviations: EDWT, maximal end-diastolic wall thickness; Max/min EDWT, maximal to minimal end-diastolic wall thickness ratio; LV, left ventricular; EF, ejection fraction; ESVi, end-systolic volume index; EDVi, end-diastolic volume index; SVi, stroke volume index; Mi, mass index; EDWT/LVEDVi, maximal end-diastolic wall thickness to left ventricular end-diastolic volume index ratio; LVM/LVEDV, left ventricular mass to left ventricular end-diastolic volume ratio evaluated using conventional (CQ) and threshold based quantification (TQ).

### Characteristics of patient and subject groups–Comparison of left ventricular CMR parameters in athletes and HCM patients evaluated using threshold-based and conventional quantification method

Both male and female HCM patients had higher EDWT compared to athletes (22.1 mm vs. 12.6 mm; 19.7 vs. 10.4, respectively), 14.8% of male HCM patients, 47.5% of male athletes, 19.8% of female HCM patients and 4.1% of female athletes were in the grey zone of borderline hypertrophy (EDWT 13–16 mm). Thirty-five percent of our male rowers, 52% of canoe and kayak paddlers and 54% of water-polo players showed EDWT between 13–16 mm. Using conventional quantification sport indices EDWT/LVEDVi_CQ_ and LVM_CQ_/LVEDV_CQ_ were significantly lower in athletes compared to HCM patients. Using threshold-based quantification method, LVMi_TQ_ was lower in male athletes compared to HCM patients, TPM and TPM% were also lower in athletes than in HCM patients. Sport indices EDWT/LVEDVi_TQ_ and LVM_TQ_/LVEDV_TQ_ were also lower in athletes compared to HCM patients. **(Table 2)**

**Table 2:** Left ventricular CMR parameters in the four subgroups evaluated using conventional (CQ) and threshold-based (TQ) quantification.

	Male athlete (n = 101)	Male HCM (n = 108)	Female athlete (n = 49)	Female HCM (n = 86)
	mean ± SD	mean ± SD	mean ± SD	mean ± SD
EDWT (mm)	12.6 ± 1.3*^¥^	22.1 ± 5.4^$^	10.4 ± 1.2^#^	19.7 ± 5.2
Max/min EDWT	1.93 ± 0.30*^¥^	3.56 ± 1.53	2.17 ± 0.45^#^	3.86 ± 1.63
Conventional quantification				
LVEF_CQ_ (%)	57.4 ± 4.36*	62.2 ± 7.29^$^	58.4 ± 4.51^#^	63.8 ± 8.31
LVESVi_CQ_ (ml/m^2^)	52.6 ± 9.60*^¥^	35.2 ± 10.1^$^	44.7 ± 7.72^#^	29.1 ± 9.67
LVEDVi_CQ_ (ml/m^2^)	123 ± 14.0*^¥^	91.6 ± 16.7^$^	107 ± 11.2^#^	79.9 ± 13.8
LVSVi_CQ_ (ml/m^2^)	73.2 ± 14.8*^¥^	57.5 ± 11.1^$^	62.5 ± 6.80	50.7 ± 9.96
LVMi_CQ_ (g/m^2^)	90.3 ± 14.7*^¥^	99.9 ± 34.0^$^	65.9 ± 10.7	76.9 ± 22.9
Max diast/LVEDVi_CQ_ (mm×m^2^/ml)	0.10 ± 0.02*	0.25 ± 0.08	0.10 ± 0.02^#^	0.25 ± 0.07
LVM_CQ_/LVEDV_CQ_ (g/ml)	0.75 ± 0.13*^¥^	1.08 ± 0.30	0.62 ± 0.10^#^	0.97 ± 0.25
Threshold-based quantification				
LVEF_TQ_ (%)	65.7 ± 4.9*	71.9 ± 9.0^$^	65.7 ± 6.4^#^	74.4 ± 8.6
LVESVi_TQ_ (ml/m^2^)	34.9 ± 7.4*^¥^	18.6 ± 6.5^$^	30.4 ± 7.1^#^	14.5 ± 6.3
LVEDVi_TQ_ (ml/m^2^)	101.0 ± 12.1*^¥^	66.2 ± 11.1^$^	89.3 ± 10.1^#^	57.0 ± 11.1
LVSVi_TQ_ (ml/m^2^)	66.3 ± 7.6*^¥^	47.6 ± 10.2^$^	58.8 ± 7.1^#^	42.3 ± 9.7
LVMi_TQ_ (g/m^2^)	113.0 ± 16.8*^¥^	126.0 ± 40.5^$^	84.3 ± 12.1^#^	101.0 ± 27.8
Max diast/LVEDVi_TQ_ (mm×m^2^/ml)	0.13 ± 0.02*	0.34 ± 0.10	0.12 ± 0.02^#^	0.36 ± 0.12
LVM_TQ_/LVEDV_TQ_ (g/ml)	1.13 ± 0.16*^¥^	1.93 ± 0.60	0.95 ± 0.14^#^	1.83 ± 0.56
TPM (g)	44.8 ± 11.5*^¥^	55.6 ± 17.5^$^	32.1 ± 7.7	40.5 ± 11.1
TPMi (g/m^2^)	21.4 ± 4.8*^¥^	27.4 ± 8.6^$^	17.7 ± 4.2	23.0 ± 6.5
TPM% = $\frac{TPM\;[g]}{LVM\;[g]}$ x 100 (%)	19.0 ± 3.7*^¥^	22.1 ± 4.6	21.1 ± 4.7	23.1 ± 4.1

Between-groups comparisons were based on least-squares linear regression if normality assumptions were satisfied, and median regression otherwise. Models were stratified by HCM when comparing males versus females, and stratified for sex when comparing HCM patients versus athletes (pairwise comparisons), adjustment for age and heart frequency was made.

*significantly different from male HCM, ^#^significantly different from female HCM, ^¥^significantly different from female athletes, ^$^significantly different from female HCM

Difference estimates adjusted for heart frequency and age. Abbreviations: EDWT, maximal end-diastolic wall thickness; Max/min EDWT, maximal to minimal end-diastolic wall thickness ratio; LV, left ventricular; EF, ejection fraction; ESVi, end-systolic volume index; EDVi, end-diastolic volume index; SVi, stroke volume index; Mi, mass index; EDWT/LVEDVi, maximal end-diastolic wall thickness to left ventricular end-diastolic volume index ratio; LVM/LVEDV, left ventricular mass to left ventricular end-diastolic volume ratio evaluated using conventional (CQ) and threshold based quantification (TQ).

### Gender-specific differences

Compared to female athletes, male athletes showed significantly higher maximal EDWT, left ventricular volumes, mass and LVM/LVEDV using both conventional and threshold-based quantification method. TPMi was also higher and TPM% was lower in male athletes, LVEF showed no significant difference between male and female athletes. **(Table 2)**

### Cut-off values and diagnostic accuracy of sport indices to differentiate HCM and athlete’s heart

**Fig 4:**
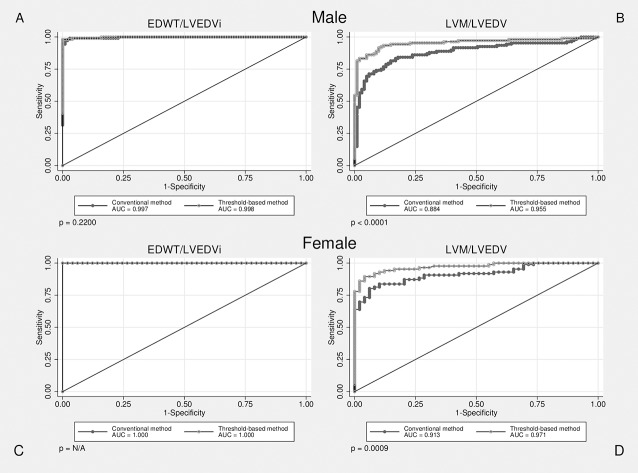
ROC curves visualizing correct identification of HCM among male and female subjects, p values represent the difference in diagnostic performance between conventional and threshold-based quantification methods.

**Fig 5:**
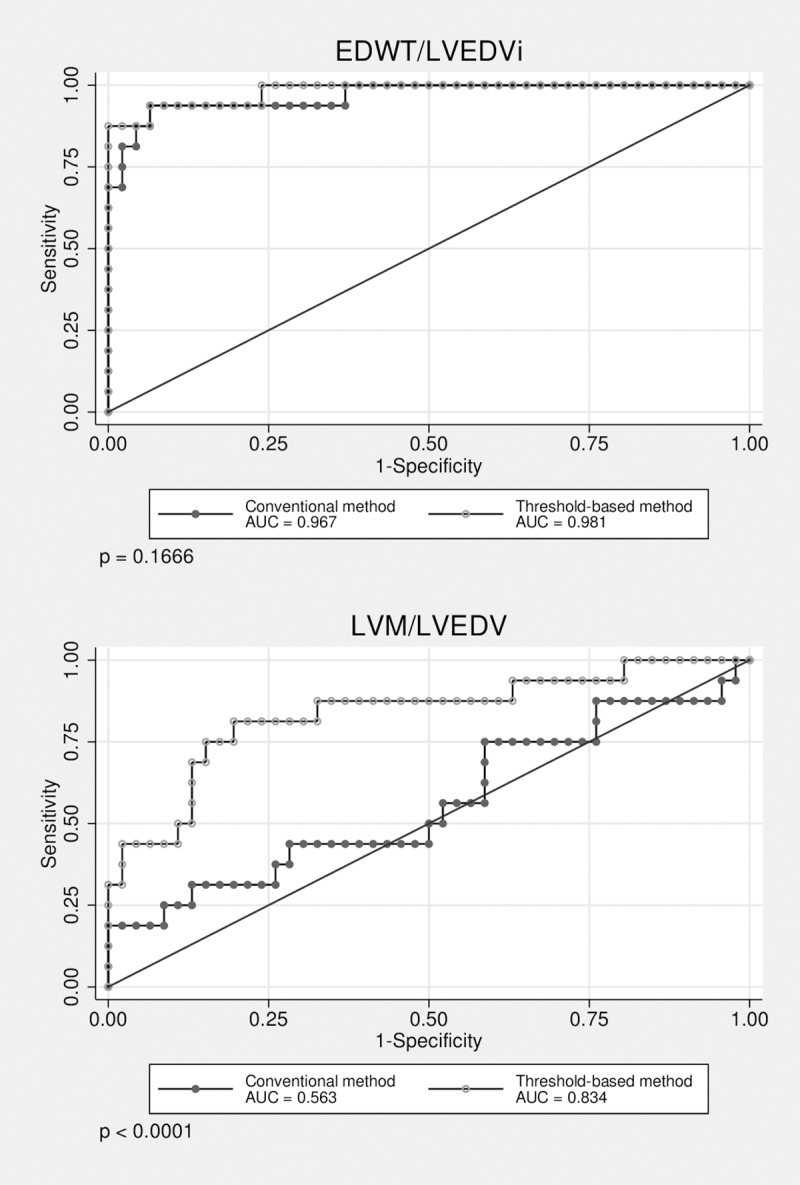
ROC curves visualizing correct identification of HCM among subgroup of individuals with EDWT between 13–16 mm, p values represent the difference in diagnostic performance between conventional and threshold-based quantification methods.

We compared the efficiency of CMR based sport indices in differentiation athlete’s heart from HCM evaluated using conventional and threshold-based methods. Although efficiency of EDWT/LVEDVi evaluated using TQ and CQ showed no significant difference, LVM/LVEDV evaluated using TQ performed significantly better than CQ in both males and females. **(Fig 4)** Both sport indices established using TQ performed significantly better than max/min EDWT (p<0.05). Cut-off value for max/min EDWT>2.4 (AUC 0.900) discriminated with a sensitivity of 81.4 and a specificity of 86.7%. Sport indices showed also high diagnostic accuracy in the male subgroup with EDWT between 13-16mm, LVM/LVEDV evaluated using TQ performed significantly better than CQ. **(Fig 5)** Cut-off value for ratio of EDWT/LVEDVi_CQ_ less than 0.14 mm×m^2^/ml and cut-off value for EDWT/LVEDVi_TQ_ less than 0.17 discriminated between physiological and pathological LV hypertrophy with a sensitivity of 99.5% and 99.0%, a specificity of 98% and 99.3%, respectively. Cut-off value for ratio of LVM/LVEDV_CQ_ less than 0.82 mm×m^2^/ml and cut-off value for LVM/LVEDV_TQ_ less than 1.27 discriminated between physiological and pathological LV hypertrophy with a sensitivity of 77.8% and 89.2%, a specificity of 86.7% and 91.3%, respectively. **(Table 3)** Despite the apparent gender-specific differences in cardiovascular sport adaptation our cut-off values regarding sport indices are applicable in both males and females.

**Table 3:** Cut-off values for optimised sensitivity and specificity.

	Cut-off value	AUC	Sensitivity	Specificity	Positive predictive value	Negative predictive value	Correctly classified instances
Conventional quantification							
EDWT/LVEDVi_CQ_ (mm×m^2^/ml)	>0.14	0.998	99.5	98.0	95.5	99.3	99.4
LVM_CQ_/LVEDV_CQ_ (g/ml)	>0.82	0.873	77.84	86.7	88.3	75.1	93.6
Threshold-based quantification							
EDWT/LVEDVi_TQ_ (mm×m^2^/ml)	>0.17	0.999	99.0	99.3	99.5	98.7	99.4
LVM_TQ_/LVEDV_TQ_ (g/ml)	>1.27	0.948	89.2	91.3	93.0	86.7	94.4

Abbreviations: EDWT/LVEDVi, maximal end-diastolic wall thickness to left ventricular end-diastolic volume index ratio; LVM/LVEDV, left ventricular mass to left ventricular end-diastolic volume ratio evaluated using conventional (CQ) and threshold based quantification (TQ).

According to our cut-off values EDWT/LVEDVi_CQ_, EDWT/LVEDVi_TQ_, LVM_CQ_/LVEDVi_CQ_ and LVM_TQ_/LVEDVi_TQ_ were in the pathological range in 9, 9, 8 and 10 athletes with HCM, respectively. **(Fig 2)**

Thirteen percent of our HCM patients or athletes and 30% of our athletes with HCM were reclassified based on EDWT/LVEDVi or LVM/LVEDV evaluated using TQ.

## Discussion

Although numerous studies have investigated the morphologic cardiac adaptation of elite athletes, we have only limited published data providing CMR based cut-off values to differentiate athlete’s heart and HCM [12, 21]. To the best of our knowledge, this study is the first to report CMR based sport indices for differentiate athlete’s heart and HCM based on a large highly trained elite athlete population applying both conventional and threshold-based quantification methods.

Increased left ventricular trabeculation observed in 18% of highly trained athletes suggests the importance of quantification of trabeculae in athletes therefore quantification of trabeculae may have an important effect on LV parameters especially in athletes [22]. A previous study has already showed an improved accuracy in evaluation of left ventricular volumes compared to aortic flow measurement as a reference and 50% reduction in time required for analysis [19]. Moreover threshold-based quantification method provides an excellent interobserver variability [18]. Besides these advantages, our study suggests that for sport index LVM_TQ_/LVEDVi_TQ_ TQ provides significantly better diagnostic accuracy than the conventional method even in subjects in the grey zone of hypertrophy.

In contrast with prior–mainly two-dimensional echocardiographic–data referring 2–15% of male athletes in the grey zone of left ventricular hypertrophy [3, 23], 47.5% of male athletes in our population reached EDWT of 13 mm. According to previous observations, gender, age, BSA, level of dynamic and static component, training hours and intensity can also have a high impact on EDWT. Being members of the National or Olympic Team, our athletes represent an elite athlete population with very intensive and regular training. All of our athletes were performing highly dynamic and at least moderately static sports, and the CMR scans were performed in competition period. These characteristics of our athlete population can be the explanation of the high rate of grey zone athletes. Moreover, literature data suggest that echocardiography may underestimate EDWT compared to CMR [24, 25]. Using echocardiography reliable delineation of LV wall significantly depends on the acoustic window, and EDWT could be underestimated because the epicardial border (especially in the LV free wall) is not visualized accurately. Moreover echocardiographic EDWT measurements are not always performed perfectly perpendicularly to the myocardial center line.

In young army recruits, 23% fall into the grey zone of hypertrophy (EDWT>13mm) measured by CMR [7], and these data suggest that high EDWT and left ventricular asymmetry could be more common in elite athletes than echocardiographic data imply. As pronounced asymmetry could be observed in highly trained healthy athletes, applying the maximal to minimal end-diastolic wall thickness ratio criteria to differentiate HCM from athlete’s heart might not be suitable. Based on our large population, sport indices established using TQ method showed significantly better diagnostic accuracy compared to maximal to minimal end-diastolic wall thickness ratio.

Although we observed that highly trained male athletes may reach the grey zone of hypertrophy in almost 50%, this pronounced cardiovascular response is absent in female athletes. Our findings indicate that gender-specific differences in cardiovascular sport adaptation are not negligible. In our study the AUC value of 1 evaluated in EDWT/LVEDVi using both conventional and threshold-based methods among female patients characterized a perfect classification result in female individuals, suggesting that differentiation between HCM and athlete’s heart in female individuals presents no remarkable difficulty.

Detection of LGE in the hypertrophic segments or in the insertion points may help the diagnosis of HCM in athletes, but lack of myocardial fibrosis does not rule out the possibility of the disease. In our study population none of the healthy athletes showed delayed enhancement, 75% of non-athlete HCM patients and only 40% of athletes with HCM showed delayed enhancement. Based on our findings LGE has an excellent positive predictive value, but low negative predictive value, in athletes with HCM even lower than in sedentary HCM patients. Compared to LGE our sport indices established using threshold-based quantification (EDWT/LVEDVi_TQ_ and LVM/LVEDV_TQ_) provide higher negative predictive values (70% vs. 99% and 87%, respectively). In the group of athletes with HCM patients without LGE the sport indices established using TQ method (EDWT/LVEDVi_TQ_ and LVM/LVEDV_TQ_) were in the pathological range in 83.3% and 100%, respectively. These findings suggest that in athletes with grey zone hypertrophy and without LGE threshold-based quantification could help in the differential diagnosis.

Recent literature data imply, that phenotype of sedentary and athletic HCM patients may differ [26], therefore collecting detailed information about highly trained athletes with HCM would be essential. Differential diagnosis could be difficult mainly in active athletes therefore we enrolled only athletes with HCM in training/competition period. Highly intensive sport performance is discouraged in HCM therefore after diagnosing HCM they usually stop or decrease the intensity of sport performance. That can be the explanation for the small number of patients in the athletic HCM group.

Although our findings are based on a large cohort, our results represent a single-centre study with all its limitations. Additionally, our HCM patients represent an older population compared to our athletes; we made an effort to eliminate the potential confounding effect of this by adjusting our estimates for age.

## Conclusions

This study highlights that elite healthy male athletes with very intensive and regular training may reach the grey zone of hypertrophy in almost 50%, which may cause diagnostic challenge in the clinical routine. However, only 4.1% of highly trained female athletes reach EDWT of 13 mm. CMR based sport indices provide an important tool to diagnose HCM and distinguish it from athlete’s heart. Not only EDWT/LVEDVi_CQ_ but also our new indices determined using TQ (EDWT/LVEDVi_TQ_ and LVM_TQ_/LVEDVi_TQ_) showed high diagnostic accuracy both in the whole patient population and in the male subgroup with EDWT 13–16 mm. In all of our athletes with HCM, the only parameter falling into the pathological range was the LVM_TQ_/LVEDV_TQ_ ratio.

## Abbreviations

AUC: Area under the receiver operating curve
BSA: body surface area
CMR: cardiac magnetic resonance
CQ: conventional quantification
EDWT: maximal end-diastolic wall thickness
EDWT/LVEDVi_CQ_: maximal end-diastolic wall thickness and left ventricular end-diastolic volume index ratio evaluated using conventional quantification
EDWT/LVEDVi_TQ_: maximal end-diastolic wall thickness and left ventricular end-diastolic volume index ratio evaluated using threshold-based quantification
ESC: European Society of Cardiology
HCM: hypertrophic cardiomyopathy
LGE: late gadolinium enhancement
LVEF_CQ_: left ventricular ejection fraction evaluated using conventional quantification
LVEF_TQ_: left ventricular ejection fraction evaluated using threshold-based quantification
LVEDVi_CQ_: left ventricular end-diastolic volume index evaluated using conventional quantification
LVEDVi_TQ_: left ventricular end-diastolic volume index evaluated using threshold-based quantification
LVESVi_CQ_: left ventricular end-systolic volume index evaluated using conventional quantification
LVESVi_TQ_: left ventricular end-systolic volume index evaluated using threshold-based quantification
LVMi_CQ_: left ventricular mass index evaluated using conventional quantification
LVMi_TQ_: left ventricular mass index evaluated using threshold-based quantification
LVM_CQ_/LVEDV_CQ_: left ventricular mass and left ventricular end-diastolic volume ratio evaluated using conventional quantification
LVM_TQ_/LVEDV_TQ_: left ventricular mass and left ventricular end-diastolic volume ratio evaluated using threshold-based quantification
TPM: left ventricular myocardial trabeculation and papillary muscles
TPMi: left ventricular trabecula and papillary muscle mass index evaluated using threshold-based quantification
TPM%: left ventricular trabecular and papillary muscle mass percent evaluated using threshold-based quantification
TQ: threshold-based quantification
